# Characterizing Early T Cell Responses in Nonhuman Primate Model of Tuberculosis

**DOI:** 10.3389/fimmu.2021.706723

**Published:** 2021-08-17

**Authors:** Riti Sharan, Dhiraj Kumar Singh, Jyothi Rengarajan, Deepak Kaushal

**Affiliations:** ^1^Southwest National Primate Research Center, Texas Biomedical Research Institute, San Antonio, TX, United States; ^2^Emory Vaccine Center and Yerkes National Primate Research Center (YNPRC), Emory University School of Medicine, Atlanta, GA, United States

**Keywords:** ESAT-6/CFP-10, T cell responses, IFN-γ, TNF-α, LTBI

## Abstract

Tuberculosis (TB), caused by *Mycobacterium tuberculosis (Mtb)*, remains a leading infectious disease killer worldwide with 1.4 million TB deaths in 2019. While the majority of infected population maintain an active control of the bacteria, a subset develops active disease leading to mortality. Effective T cell responses are critical to TB immunity with CD4^+^ and CD8^+^ T cells being key players of defense. These early cellular responses to TB infection have not yet been studied in-depth in either humans or preclinical animal models. Characterizing early T cell responses in a physiologically relevant preclinical model can provide valuable understanding of the factors that control disease development. We studied *Mtb*-specific T cell responses in the lung compartment of rhesus macaques infected with either a low- or a high-dose of *Mtb* CDC1551 *via* aerosol. Relative to baseline, significantly higher *Mtb*-specific CD4^+^IFN-γ^+^ and TNF-*α* ^+^ T cell responses were observed in the BAL of low dose infected macaques as early as week 1 post TB infection. The IFN-γ and TNF-*a* response was delayed to week 3 post infection in *Mtb*-specific CD4^+^ and CD8^+^T cells in the high dose group. The manifestation of earlier T cell responses in the group exposed to the lower *Mtb* dose suggested a critical role of these cytokines in the antimycobacterial immune cascade, and specifically in the granuloma formation to contain the bacteria. However, a similar increase was not reflected in the CD4^+^ and CD8^+^IL-17^+^ T cells at week 1 post infection in the low dose group. This could be attributed to either a suppression of the IL-17 response or a lack of induction at this early stage of infection. On the contrary, there was a significantly higher IL-17^+^ response in *Mtb*-specific CD4^+^ and CD8^+^T cells at week 3 in the high dose group. The results clearly demonstrate an early differentiation in the immunity following low dose and high dose infection, largely represented by differences in the IFN-γ and TNF-α response by *Mtb*-specific T cells in the BAL. This early response to antigen expression by the bacteria could be critical for both bacterial growth control and bacterial containment.

## Introduction

Tuberculosis (TB) remains the leading cause of human death from a single infectious agent with a total of 1.4 million deaths in 2019 (1). The outcome of a pulmonary TB infection can either be complete clearance of the pathogen to active tuberculosis (ATB) disease. The percentage of the infected population developing the clinical symptoms of TB remains small with a much higher percentage being able to control the naturally acquired infections (2, 3). This latently infected population largely remains asymptomatic and in some cases even clear the infections (4). Generation of robust T cell responses is critical in the immunity to TB and are responsible for a dynamic balance between the host and pathogen in a latent TB infection (LTBI) (5). While co-morbidities, such as, with HIV is a known factor for the reactivation of LTBI (6), the underlying causes for the susceptibility to the active disease remains unknown. Antigen specific responses to TB infection, including novel features of T cell differentiation have revealed pathways that facilitate the immune control of infection (7). The production of inflammatory cytokines such as gamma interferon (IFN-γ) and tumor necrosis factor alpha (TNF-α) are critical in the protection against long-term rampant *Mtb* growth and loss of these factors leads to heightened *Mycobacterium tuberculosis* (*Mtb)* replication and death (8, 9). Indeed, stimulation with *Mtb* antigens Early Secretory Antigenic Target (ESAT)-6 and Culture Filtrate Protein (CFP)-10 induces IFN-γ and TNF-α production by the CD4^+^ and CD8^+^T cells that may provide tools to study the role of these early responses in protection from a fatal infection.

Characterizing the phenotype and function of these early T cell responses could provide a critical tool to distinguishing latent from active TB disease in future experiments wherein, the macaques would be followed for a longer duration of time (10). The aim of this study is to characterize the early T cell responses in a nonhuman primate (NHP) model of TB. The model recapitulates humans, wherein, the infectious doses differ between individuals. There have been reports of differential impact on functional CD4^+^ and CD8^+^ T cell responses by the disease stage and bacterial burden (11–13). However, there is a paucity of data on the distinguished early adaptive response signatures in a biologically and physiologically relevant animal model. The NHP model of TB serves as an excellent model recapitulating the spectrum of immune responses observed in humans, including the pathology (14, 15). Manipulating the bacteria in a macaque model of TB infection presents a valuable tool to dissect the local immune responses in a TB predominant microenvironment that is not possible in any other animal model (16–18). We hypothesized that measuring the TB-specific T cell responses early in a rhesus macaque model of TB infection could provide a better understanding of the early responses and their potential role in disease progression. Hence, we performed high parameter flow cytometry on stimulated bronchoalveolar lavage (BAL) cells from macaques infected *via* aerosol, with a low dose and high dose of *Mtb*, to measure key cytokines in TB infection, IFN-γ, TNF-α and IL-17 produced by CD4^+^ and CD8^+^ T cells in response to ESAT-6/CFP-10 and *Mtb* Cell Wall Fraction (*Mtb* CW). This enabled a comprehensive elucidation of the differences in the early responses and provided a potential tool to delineate the disease progression in long-term studies.

## Materials and Methods

### Study Approval

All infected animals were housed under Animal Biosafety Level 3 facilities at the Southwest National Primate Research Center, where they were treated according to the standards recommended by AAALAC International and the NIH guide for the Care and Use of Laboratory Animals. The study procedures were approved by the Animal Care and Use Committee of the Texas Biomedical Research Institute.

### Animal Infections

**Figure 1:**
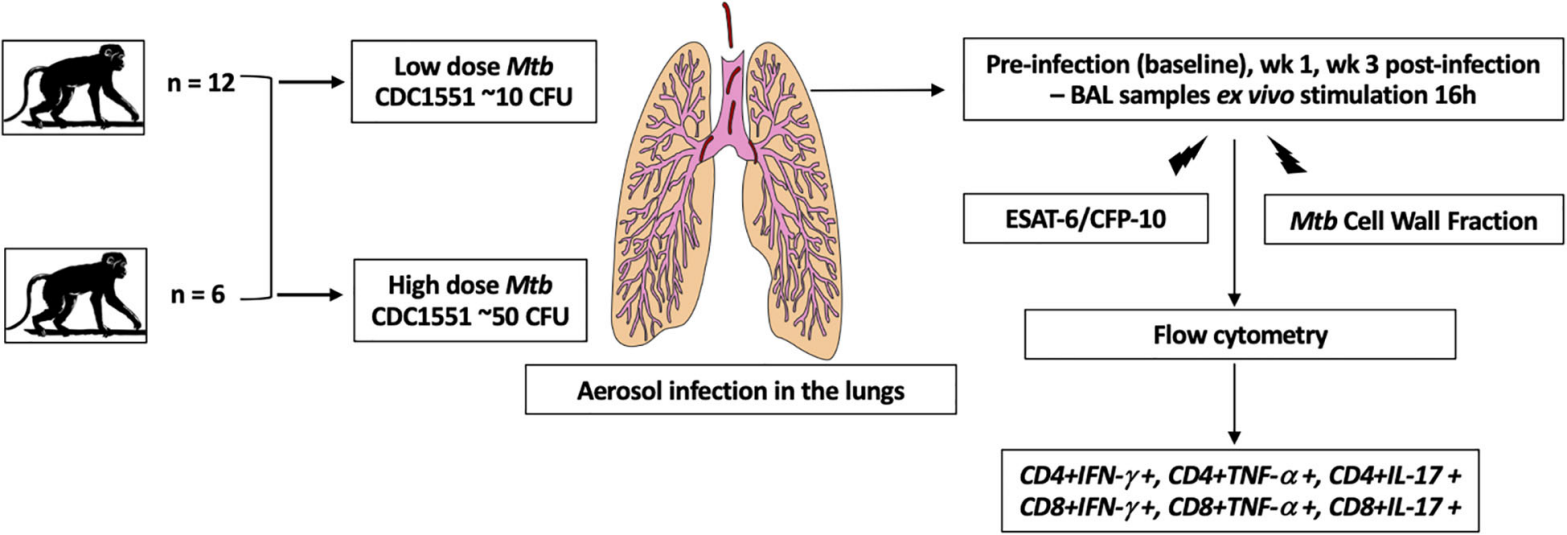
Schematic of the study design. We infected 2 groups of adult Indian rhesus macaques with *Mtb* CDC1551 *via* aerosol. The first group (*n* = 12) had a low dose of approximately 10 CFU deposited in the lungs while the second group (*n* = 6) had a higher dose of 50 CFU deposited in the lungs. The BAL cells were collected at pre-infection, wk 1 and 3 post-infection. They were stimulated *ex vivo* with *Mtb*-specific antigens, ESAT-6/CFP-10 and *Mtb* Cell Wall Fraction. After stimulation, the cells were stained with the surface antibodies for flow cytometry and acquired on BD Symphony. Analysis was performed using FlowJo (v10.6.1).

The study design is outlined in **Figure 1**. We infected 2 groups of specific pathogen free adult Indian rhesus macaques from the SNPRC colony with *Mtb* CDC1551 *via* aerosol. The first group (n=12) had a low dose of approximately 10 CFU deposited in the lungs while the second group (n=6) had a higher dose of 50 CFU deposited in the lungs. All higher dose infected animals had a positive tuberculin skin test 3 weeks after exposure, while the low dose infected group were TST positive at 5 weeks, confirming infection. The animals were monitored for C-Reactive Protein (CRP) values (an acute phase protein and inflammatory marker), body temperatures and body weights.

### Antigen Stimulations and Flow Cytometry

The freshly collected BAL cells were stimulated *ex vivo* with *Mtb*-specific antigens, ESAT-6/CFP-10 and *Mtb* Cell Wall Fraction (BEI Resources, 10 μg/mL) for a total of 16 h. Brefeldin A (0.5 μg/mL, SIGMA) was added 2 h after the onset of stimulation. After stimulation, the cells were stained with LIVE/DEAD fixable Near-IR stain (ThermoFisher) and stained subsequently with the surface antibodies: CD4-PerCP-Cy5.5 (L200, BD Biosciences), CD8-APC (RPA, T8, BD Biosciences), CD3-AlexaFlour 700 (SP34 2, BD Biosciences), CD95-BV421 (DX2, BD Biosciences), CD28-PECy7 (CD28.2, BD Biosciences) and CD45-BUV395 (D058 1283, BD Biosciences). Cells were then fixed, permeabilized and stained with intracellular antibodies: IFNγ-APC-Cy7 (B27, Biolegend), IL-17-BV605 (BL168, Biolegend) and TNF-α-BV650 (MAb11, Biolegend). Cells were washed, suspended in BD stabilizing fixative buffer and acquired on BD Symphony flow cytometer. Analysis was performed using FlowJo (v10.6.1) using previously published gating strategy (18–20) (**Figures S1** **–** **S3**).

### Statistical Analysis

Statistical analysis was performed using GraphPad Prism (version 8.4.1). Significance was determined using Mann Whitney U test in GraphPad Prism v8.4.1. A *P* value of <0.05 was considered as statistically significant. *P < 0.05; **P <0.01; ***P < 0.001; ****P < 0.0001. Data are represented as median with interquartile range.

## Results

### Clinical Parameters

**Figure 2:**
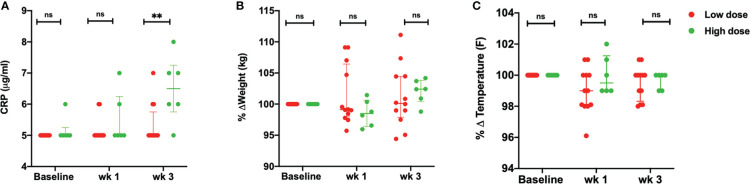
Clinical parameters. **(A)** Serum CRP values (µg/mL) **(B)** percentage weight change (kg) and **(C)** percentage body temperature change (°F) of low dose (*n* = 12) and high dose (*n* = 6) *Mtb* infected rhesus macaques at baseline, wk 1 and 3 post-infection. The data are expressed as median with interquartile range. **P < 0.01; ns, non significant. Significance was determined using Mann Whitney U test in GraphPad Prism v8.4.1.

Upon infection with the low dose of *Mtb*, did not demonstrate the clinical signs of disease. These animals maintained low CRP values with not more than 5-7% body weight loss or fever (**Figure 2A**). Viable bacilli were not readily detected in the BAL of these animals (data not shown). On the contrary, the animals that received a high dose of 50 CFU, displayed higher than baseline CRP values (> 5 µg/mL) as early as 3 weeks post infection. No significant changes were observed in the body weight (**Figure 2B**) and temperature (**Figure 2C**) of this group up till week 3 of infection.

### Early Mtb-Specific CD4^+^ IFN-γ and TNF-α Response in Low Dose Infected Macaques

**Figure 3:**
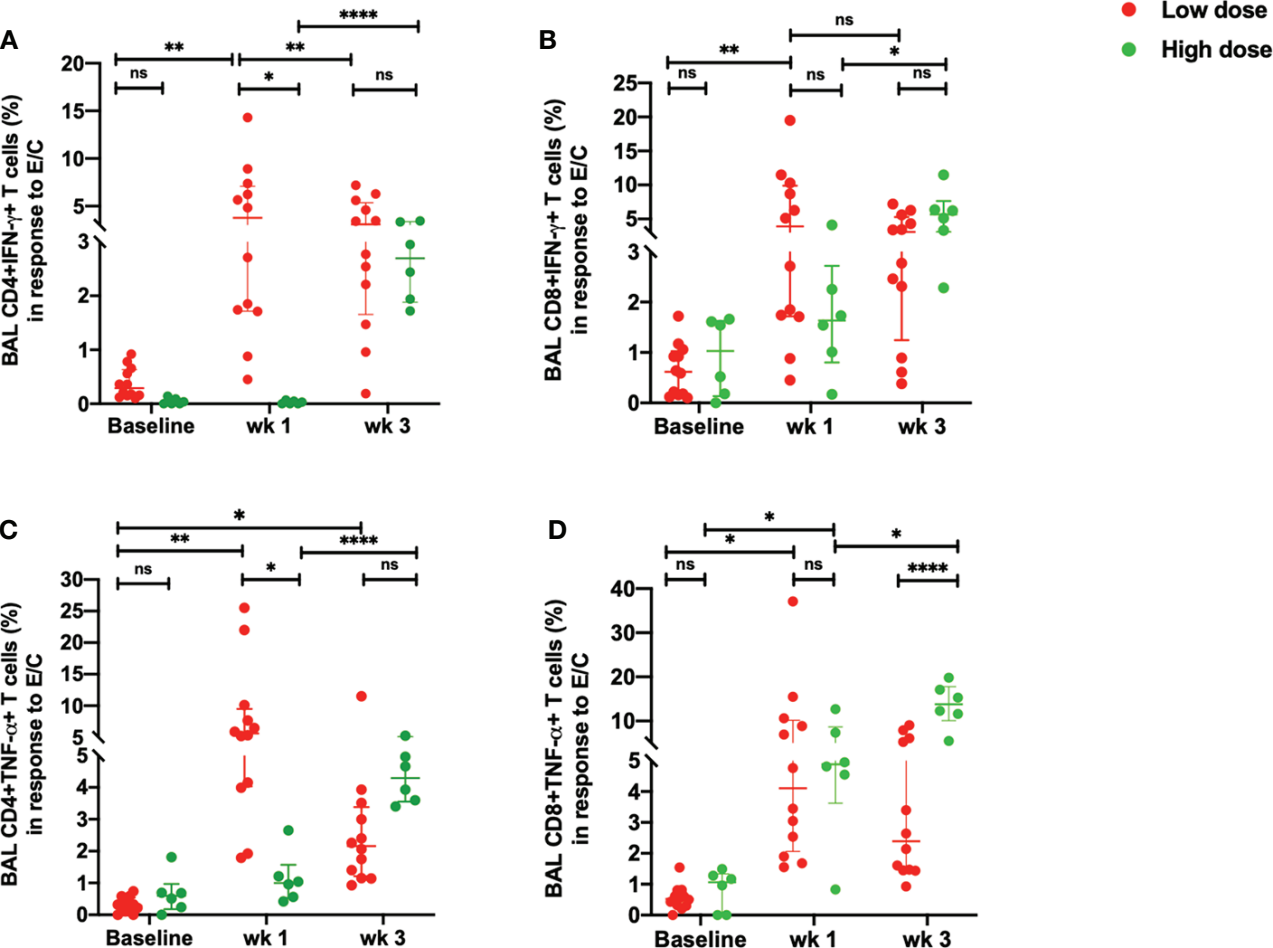
Early ESAT-6/CFP-10-specific responses in the BAL. **(A)** percentage of CD4+IFN-γ+ T cells, **(B)** percentage of CD8+IFN-γ+ T cells, **(C)** percentage of CD4+TNF-α+ T cells and **(D)** percentage of CD8+ TNF-α+ T cells in response to ESAT/6/CFP-10 stimulation in low dose (*n* = 12) and high dose (*n* = 6) infection. The data are expressed as median with interquartile range. *P < 0.05; **P < 0.01; ****P < 0.0001; ns, non significant. Significance was determined using Mann Whitney U test in GraphPad Prism v8.4.1.

BAL samples were collected from study macaques at pre-infection, week 1 and week 3 post infection using standard operating procedures by the veterinarian. The single cells were prepared as per the lab standardized protocol (21). All *Mtb*-specific responses are background corrected. Upon stimulation with ESAT-6/CFP-10, there was a delayed IFN-γ response in the *Mtb*-specific CD4^+^T cells in the high dose compared to the low dose group (**Figure 3A**). This difference was however, not observed in the *Mtb*-specific CD8^+^T cells (**Figure 3B**). While the low dose infection resulted in a significant increase in the percentage of *Mtb*-specific CD4^+^IFN-γ^+^T cells as early as week 1 post-infection, this response was not observed in the high dose group till 3 weeks post infection (**Figure 3A**). The early response observed in the low dose infection decreased from week 1 to week 3 post-infection whereas the response spiked in the high dose infection group at week 3 post-infection (**Figure 3A**).

Similarly, there was a delayed increase in the percentage of *Mtb*-specific CD4^+^TNF-α^+^T cells in the high dose infection group with a higher percentage of this subset observed at week 3 post-infection (**Figure 3C**). On the contrary, the low dose infected macaques demonstrated an early TNF-α response in the *Mtb*-specific CD4^+^ T cells at weeks 1 which decreased at week 3 post-infection (**Figure 3C**). CD4^+^TNF-α^+^T cells were significantly higher in the low dose group than the high dose group at week 1 post-infection. Similarly, *Mtb*-specific CD8^+^TNF-α^+^T cells exhibited a significant increase in the high dose group at 3 weeks post-infection compared to the low dose infection group (**Figure 3D**). The low dose infection group maintained a consistent increase in the CD8^+^TNF-α^+^T cells at 1- and 3-weeks post-infection compared to the pre-infection levels (**Figure 3D**).

**Figure 4:**
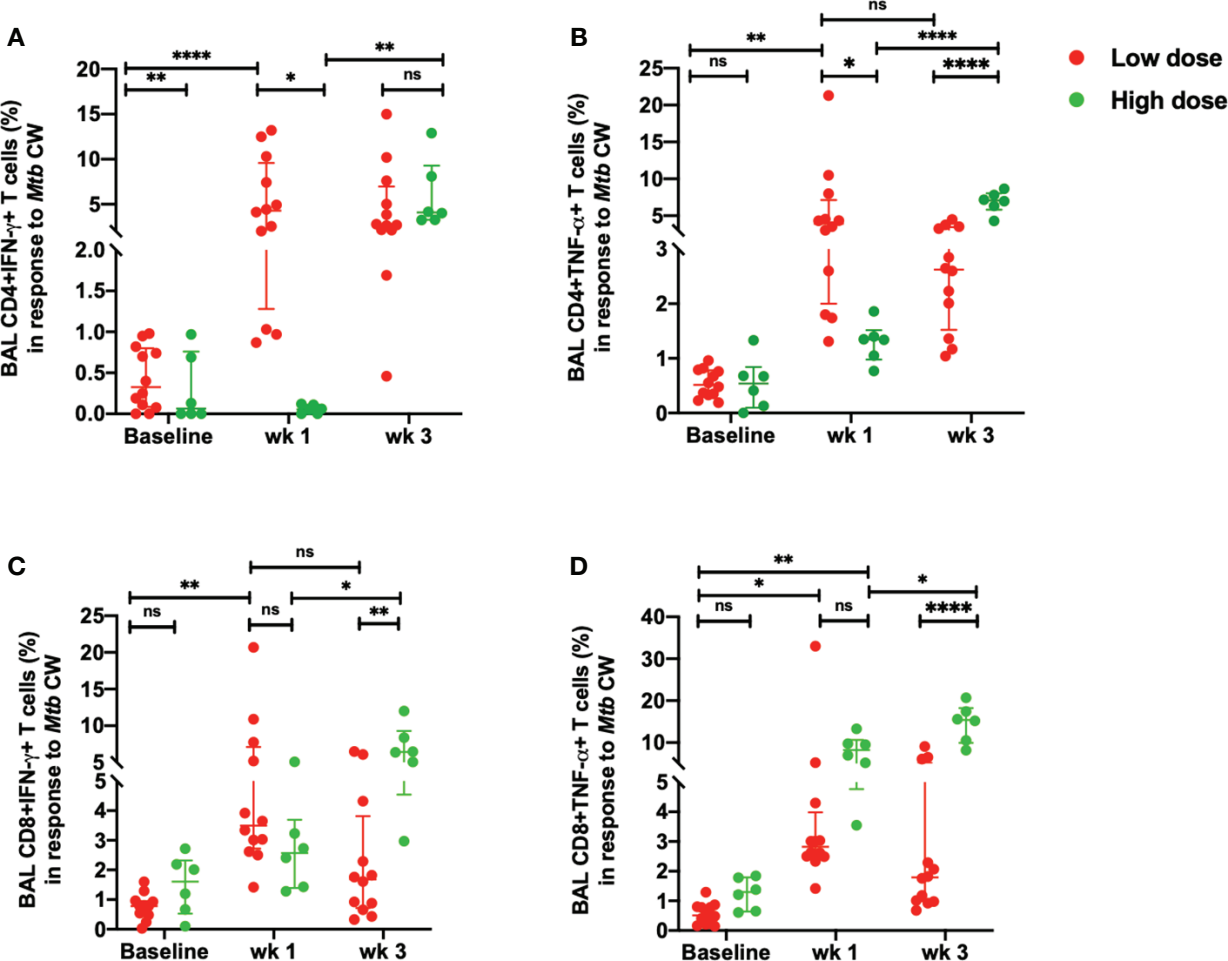
Early *Mtb* CW-specific responses in the BAL. **(A)** percentage of CD4+IFN-γ+ T cells, **(B)** percentage of CD4+TNF-α+ T cells, **(C)** percentage of CD8+ IFN-γ+ T cells and **(D)** percentage of CD8+ TNF-α+ T cells in response to *Mtb* CW stimulation in low dose (*n* = 12) and high dose (*n* = 6) infection. The data are expressed as median with interquartile range. *P < 0.05; **P < 0.01; ****P < 0.0001; ns, non significant. Significance was determined using Mann Whitney U test in GraphPad Prism v8.4.1.

When BAL cells were stimulated with *Mtb* CW, the differences observed between low dose and high dose were similar to those elicited with ESAT-6/CFP-10. Thus, the percentages of CD4+IFN-γ+ (**Figure 4A**) and CD4^+^ TNF-α^+^T cells (**Figure 4B**) were significantly lower in the high dose group compared to the low dose group at week 1 post-infection. No significant difference was seen in the IFN-γ response in the *Mtb* CW-specific CD4^+^T cells between high dose and low dose infection group at week 3 post-infection (**Figure 4A**). Similarly, a delayed IFN-γ response in the CD8^+^T cells in response to the *Mtb* CW was observed with a significant increase in the high dose infection group compared to the low dose group at 3 weeks post-infection (**Figure 4C**). As with the gamma response, the *Mtb*-specific CD4^+^TNF- α^+^T cells (**Figure 4B**) and CD8^+^ TNF-α^+^T cells (**Figure 4D**) elicited by *Mtb* CW stimulation at 3 weeks post-infection was significantly higher in the high dose group compared to the low dose group. Thus, an early and consistent TNF-α response was observed in the low dose group while a delayed but a more robust TNF-α response in both *Mtb*-specific CD4^+^ and CD8^+^T cells was observed in the high infection dose. No significant changes were observed in the unstimulated samples between the two doses (**Figures S4A, B, D, E**).

In addition to the percentage of CD4^+^ and CD8^+^ T cells positive for cytokine production, we also gated for the percentage of *Mtb*-specific T cells expressing surface phenotypic markers consistent with central memory T cells (Tcm CD28^+^CD95^+^) and effector memory T cells (Tem CD28^-^CD95^+^) in the total *Mtb*-specific CD4 and CD8 population in low dose infected animals (**Figure S5**). We observed a higher central memory (>75%) CD4^+^ T cells in response to stimulation, both in the low dose (**Figures S5A, B**) and high dose (**Figure S6**) infection. In comparison, the effector memory response was less than 20% at pre-infection, wks 1 and 3 post-infection in both the doses (**Figures S5A, B** and **S6A, B**). There were no significant differences in the percentages of Tcm and Tem from baseline to wk 1 and from wk 1 to wk 3 post-infection in response to stimulation with ESAT-6/CFP-10 and *Mtb CW* in the both the doses (**Figures S5A, B** and **S6A, B**). Comparable *Mtb*-specific central (~40%) and effector memory (~50%) CD8^+^ T cells were observed in both the doses with no significant changes from pre-infection to wk 1 and from wk 1 to wk 3 post-infection (**Figures S5C, D** and **S6C, D**).

### Controlled Early Inflammatory Response in Low Dose Mtb Infection

**Figure 5:**
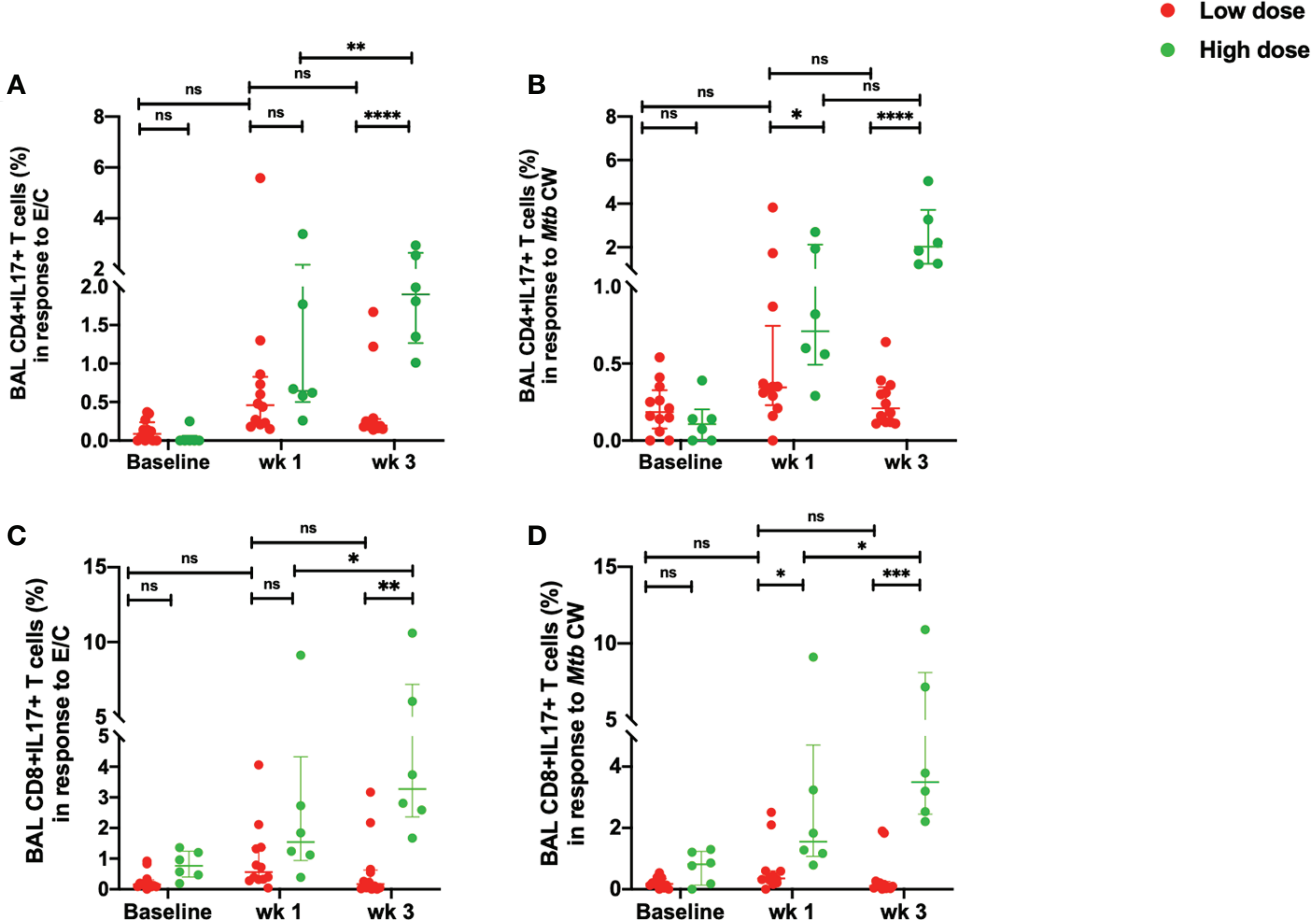
Early *Mtb*-specific IL-17 responses in the BAL. **(A)** percentage of CD4+IL-17+ T cells in response to ESAT/6/CFP-10 stimulation, **(B)** percentage of CD4+IL-17+ T cells in response to *Mtb* CW stimulation, **(C)** percentage of CD8+ IL-17+ T cells in response to ESAT/6/CFP-10 stimulation and **(D)** percentage of CD8+ IL-17+ T cells in response to *Mtb* CW stimulation in low dose (*n* = 12) and high dose (*n* = 6) infection. The data are expressed as median with interquartile range. *P < 0.05; **P <0.01; ***P < 0.001; ****P < 0.0001; ns, non significant. Significance was determined using Mann Whitney U test in GraphPad Prism v8.4.1.

There was a significant increase in the percentage of *Mtb*-specific CD4^+^ IL-17^+^T cells in the high dose infected group at week 3 compared to the low dose infected group in response to both, ESAT-6/CFP-10 and *Mtb* CW antigens (**Figures 5A, B**). The low dose infected group demonstrated a consistent measure of the CD4^+^ IL-17^+^T cells from week 1 to week 3 post-infection with no significant changes compared to the pre-infection levels (**Figures 5A, B**). Similarly, the percentage of IL-17^+^ CD8^+^T cells in response to *Mtb* CW stimulation was significantly higher in the high dose infection group compared to the low dose infection group at 3 weeks post-infection (**Figures 5C, D**). No significant changes were observed in the unstimulated samples between the two doses (**Figures S4C, F**).

## Discussion

Our results clearly outline the differences in the early *Mtb*-specific T cells responses in a low dose *versus* higher dose infection in a rhesus macaque model of TB. The macaques exposed to a low-dose controlled *Mtb* infection were associated with an early IFN-γ and TNF-α response in *Mtb*-specific CD4+ T cells. A high dose infection caused a significantly higher TNF-α response in the CD8^+^ T cells at 3 weeks post-infection but no noticeable changes in the IFN-γ response this early in infection. TNF-α secreting *Mtb*-specific CD4^+^ T cells are a promising candidate to differentiate between active and latent TB infections (10, 22). In the study by Harari et al. (22), significant increase in the proportions of *Mtb*-specific CD4^+^T cells expressing TNF-α was seen in patients with active disease and proposed to be the strongest predictor of diagnosis of active disease. Indeed, commensurate with these findings, we observed a significantly higher TNF-α response in the *Mtb*-specific CD8^+^ T cells in the group infected with a higher number of bacilli. The difference in our study was that here we compared two different doses of infection of *Mtb* in a biologically relevant animal model. Though the difference between TNF-α expression by CD4^+^ T cells was not significantly different between low dose and high dose infection groups at week 3, there was a consistent increase in the TNF-α expression from pre-infection to week 3 in the high dose group. Hence, while the low dose elicits an earlier TNF-α response that then remains at similar levels up till 3 weeks post infection, the same response is slower to develop in the higher dose but more robust as the infection progresses. Previous studies have shown the detection of *Mtb*-specific effector CD4^+^ T cells expressing IFN-γ and/or TNF-α can distinguish between a latent TB and active TB infection (12, 23). A recent study demonstrated that increased amounts of TNF-α in an active TB infection subverted the immune-surveillance by perturbing dendritic cell mediated antigen transportation to the lymph node allowing bacterial reserve (24). Further studies on phenotyping the subsets in our study to distinguish the effector and memory functions could provide a highly discriminatory readout.

IFN-γ producing CD4^+^T cells are the cornerstone of protective immunity in pulmonary *Mtb* infections (25). In the two doses studied here, the difference in the CD4^+^IFN-γ^+^ response to *Mtb* antigens, ESAT-6/CFP-10 and *Mtb* CW, was the highest at 1-week post-infection and diminished by week 3 post-infection. IFN-γ deficient mice studies have demonstrated a lack of survival even in low-dose *Mtb* infections with progression to active disease (26, 27). This early gamma response in the low dose infection alone could be representative of the protective role of CD4^+^ T lymphocytes in mediating macrophage activation *via* iNOS expression (27, 28). IFN-γ is known to promote iNOS expression in macrophages that in turn serves to recruit other reactive nitrogen intermediates (RNI) (29). Not only is this early gamma response critical for TB control, it also plays a role in the long-term survival of the host by working synergistically with the early TNF-α responses and thus contributing to the granuloma formation that controls the disease progression (30). Interestingly, we observed a significantly higher CD8^+^IFN-γ^+^ T cells in the high dose group in response to stimulation with *Mtb* CW at 3 weeks post-infection, but did not see a similar response to ESAT-6/CFP-10 stimulation. While the role of CD4^+^T cells in IFN-γ production in TB is well documented, the role of CD8^+^ T cells in the IFN-γ production in human TB is less well studied. A part of the role of the CD8^+^ T cells has been elucidated in mice experiments, wherein, mice deficient in CD8^+^ T cells were unable to control *Mtb* infection (31). Additionally, CD8^+^ T cells have been shown to undergo phenotypic and functional changes, comparable to CD4^+^ T cells during pulmonary *Mtb* infection (32). *Mtb*-specific CD8^+^ T cells have demonstrated differences in prevalence, frequency, phenotypic and functional profiles in latent *versus* active TB disease (33). Similar to our findings, a higher *Mtb*-specific CD8^+^ T cells frequency (60%) was observed in the TB patients compared to 15% in LTBI patients. These CD8^+^ T cell responses were directed against ESAT-6/CFP-10 *in vitro* stimulation comparable to our study in NHP model. Also, the IFN-γ response in the *Mtb*-specific CD8^+^ T cells was not very different between active and LTBI cases like our study, in which we did not observe a significant difference in the CD8^+^IFN-γ^+^ T cells in the low dose and high dose when stimulated with ESAT-6/CFP-10.

While Th1 cells plays a distinct role in rendering protection in TB *via* production of IFN-γ and activating antimicrobial action in macrophages (34), Th17 cells implements neutrophilic inflammation, tissue damage and TB pathology (35). The data on the role of Th17 cells in TB remains controversial with some groups reporting a higher frequency correlating with TB protection in latent patients (36) while others reported lower expression in latent patients and increased frequencies in active or multi-drug resistant patients (37–39). Some are of the verdict that Th17 cells are minimally expressed in TB and do not have a significant role to play in the protection and/or pathology of TB in humans (40, 41). In our study, we observed a significant increase in the IL-17 expressing *Mtb*-specific CD4^+^ and CD8^+^ T cells in the high dose infection compared to the low dose at 3 weeks post-infection. *Mtb* infection in humans induces IFN-γ and IL-17 and the main source is the CD4^+^IFN-γ^+^IL-17^+^ T cells (38). Moreover, the antigen-expanded CD4^+^IL-17^+^ T cells correlates with the clinical parameters associated with disease severity. Given these findings, the expansion of *Mtb*-specific CD4^+^IFN-γ^+^IL-17^+^ T cells has been proposed as a biomarker for prediction of clinical outcome in active TB patients (38). T cells from MDR-TB patients has been shown to express high levels of IL-17 *via* the strong TLR-2 dependent TGFβ production by antigen-presenting cells (37). Mouse studies mimicking human vaccination post *Mtb*-exposure verified the presence of increased IL-17 which correlated to lung tissue damage (42). Conversely, protective role of Th17 responses have also been reported in the lung tissue following BCG vaccination (43, 44). However, it is to be noted that it is feasible to observe an increased bacterial burden with a higher initial inoculum that could impact the disease kinetics. While this study aims to identify the very early differences in the adaptive response to *Mtb*, it will be critical to follow the kinetics over a longer duration in future studies to ascertain the true role of IL-17 in this model. Overall, we have demonstrated a distinct phenotype of *Mtb*-specific CD4^+^ and CD8^+^T cells following *in vitro* stimulation with ESAT-6/CFP-10 and *Mtb* CW early in TB infection in a biologically and physiologically relevant animal model. Further, in depth phenotyping of these subsets into tissue resident memory cells at later time point in future studies would prove instrumental in improving our understanding of these early T cells responses and their correlation to disease progression.

## Data Availability Statement

The original contributions presented in the study are included in the article/**Supplementary Material**. Further inquiries can be directed to the corresponding author.

## Ethics Statement

The animal study was reviewed and approved by Texas Biomedical Research Institute IACUC.

## Author Contributions

RS, DS, JR, and DK designed the study. RS and DS executed the experiments and analyzed the data. RS and DK wrote the manuscript. All authors contributed to the article and approved the submitted version.

## Funding

This work was primarily supported by NIH grants R01AI111943 and R01AI123047 (to DK and JR), 1 K01 OD031898-01 (to RS) with additional support from NIH grants R01AI111914, R01AI134240, R01AI138587, and U19AI111211 and institutional grants from the Office of the Director, NIH P51OD011133 (to SNPRC), P30 RR00165 and P51OD011132 (to YNPRC), and P30 AI050409 [Emory University Center for AIDS Research (CFAR)].

## Conflict of Interest

The authors declare that the research was conducted in the absence of any commercial or financial relationships that could be construed as a potential conflict of interest.

## Publisher’s Note

All claims expressed in this article are solely those of the authors and do not necessarily represent those of their affiliated organizations, or those of the publisher, the editors and the reviewers. Any product that may be evaluated in this article, or claim that may be made by its manufacturer, is not guaranteed or endorsed by the publisher.

## Supplementary Material

Supplementary Figure 1 Gating strategy for *Mtb*-specific responses. The cells are gated on CD45 and Live/Dead to select live cells and perform red blood cell (RBC) discrimination. This is followed by singlet gating on SSC and FSC -Area, width and Height. Total CD4 and CD8 is then gated on total CD3 population. IFN-γ+, TNF-α+ and IL-17+ CD4+ and CD8+ T cells are then gated on total CD4 and CD8 population.

Supplementary Figure 2 Gating strategy for *Mtb*-specific central memory and effector memory T cell responses. The cells are gated on CD45 and Live/Dead to select live cells and perform red blood cell (RBC) discrimination. This is followed by singlet gating on SSC and FSC -Area, width and Height. Total CD4 and CD8 is then gated on total CD3 population. Central (CD28+CD95+) and effector (CD28-CD95+) memory T cells are then gated on total CD4 and CD8 population in BAL and PBMCs.

Supplementary Figure 3 Gating strategy for *Mtb*-specific cytokine positive cells in unstimulated, ESAT-6/CFP-10 stimulated and Mtb CW stimulated BAL samples. **(A)** CD4+IFN-γ+ T cells **(B)** CD8+ IFN-γ+ T cells **(C)** CD4+IL-17+T cells **(D)** CD8+IL-17+T cells **(E)** CD4+TNF-α+T cells and **(F)** CD8+TNF-α+T cells.

Supplementary Figure 4 Unstimulated responses in BAL of low dose (n = 12) and high dose (n = 6) infected macaques. **(A)** percentage of CD4+IFN-γ+ T cells, **(B)** percentage of CD4+TNF-α+ T cells, **(C)** percentage of CD4+IL-17+ T cells, **(D)** percentage of CD8+ IFN-γ+ T cells, **(E)** percentage of CD8+TNF-α+ T cells, **(F)** percentage of CD8+IL-17+ T cells. The data are expressed as median with interquartile range. **P* < 0.05; ***P < *0.01; ****P* < 0.001; *****P* < 0.0001. Significance was determined using Mann Whitney U test in GraphPad Prism v8.4.1.

Supplementary Figure 5 Total CD4+ central and Effector memory T cell response in BAL of low dose infection (*n* = 12). **(A)** CD4+Tcm and Tem in response to ESAT-6/CFP-10 stimulation, **(B)** CD4+Tcm and Tem in response to *Mtb* CW stimulation, **(C)** CD8+ Tcm and Tem in response to ESAT-6/CFP-10 stimulation. The data are expressed as median with interquartile range. **P* < 0.05; ***P < *0.01; ****P* < 0.001; *****P* < 0.0001. Significance was determined using Mann Whitney U test in GraphPad Prism v8.4.1.

Supplementary Figure 6 Total CD4+ central and Effector memory T cell response in BAL of high dose infection (*n* = 12). **(A)** CD4+Tcm and Tem in response to ESAT-6/CFP-10 stimulation, **(B)** CD4+Tcm and Tem in response to *Mtb* CW stimulation, **(C)** CD8+ Tcm and Tem in response to ESAT-6/CFP-10 stimulation and **(D)** CD8+ Tcm and Tem in response to *Mtb* CW stimulation. The data are expressed as median with interquartile range. **P* < 0.05; ***P < *0.01; ****P* < 0.001; *****P* < 0.0001. Significance was determined using Mann Whitney U test in GraphPad Prism v8.4.1.
