# Supplementary figures and images for: Characterizing Early T Cell Responses in Nonhuman Primate Model of Tuberculosis
Source: Front Immunol. 2021 Aug 17;12:706723. doi: 10.3389/fimmu.2021.706723 (PMC8416058; doi:10.3389/fimmu.2021.706723)

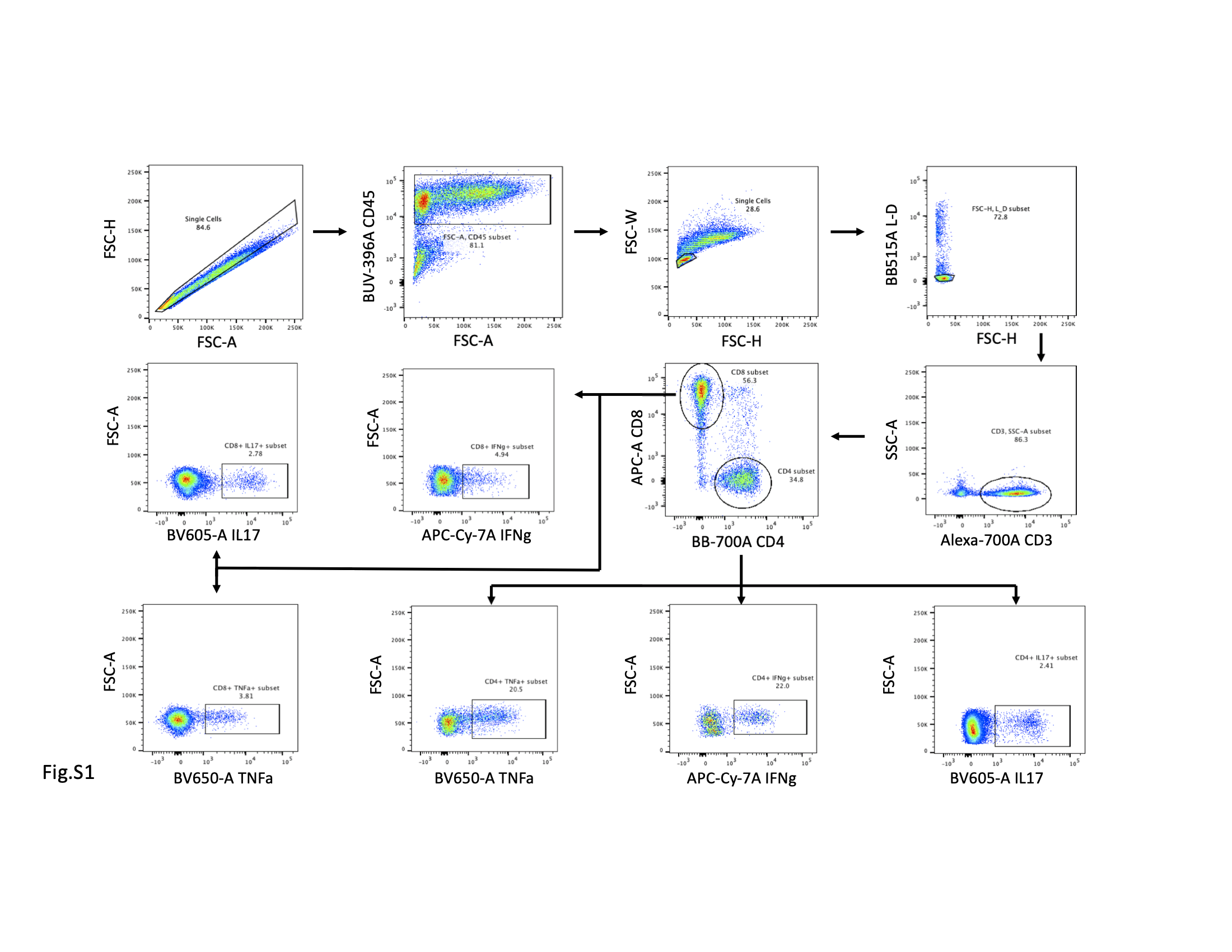

Supplement: Supplementary Figure 1 — Gating strategy for Mtb-specific responses. The cells are gated on CD45 and Live/Dead to select live cells and perform red blood cell (RBC) discrimination. This is followed by singlet gating on SSC and FSC -Area, width and Height. Total CD4 and CD8 is then gated on total CD3 population. IFN-γ+, TNF-α+ and IL-17+ CD4+ and CD8+ T cells are then gated on total CD4 and CD8 population. [file Image_1.tiff]

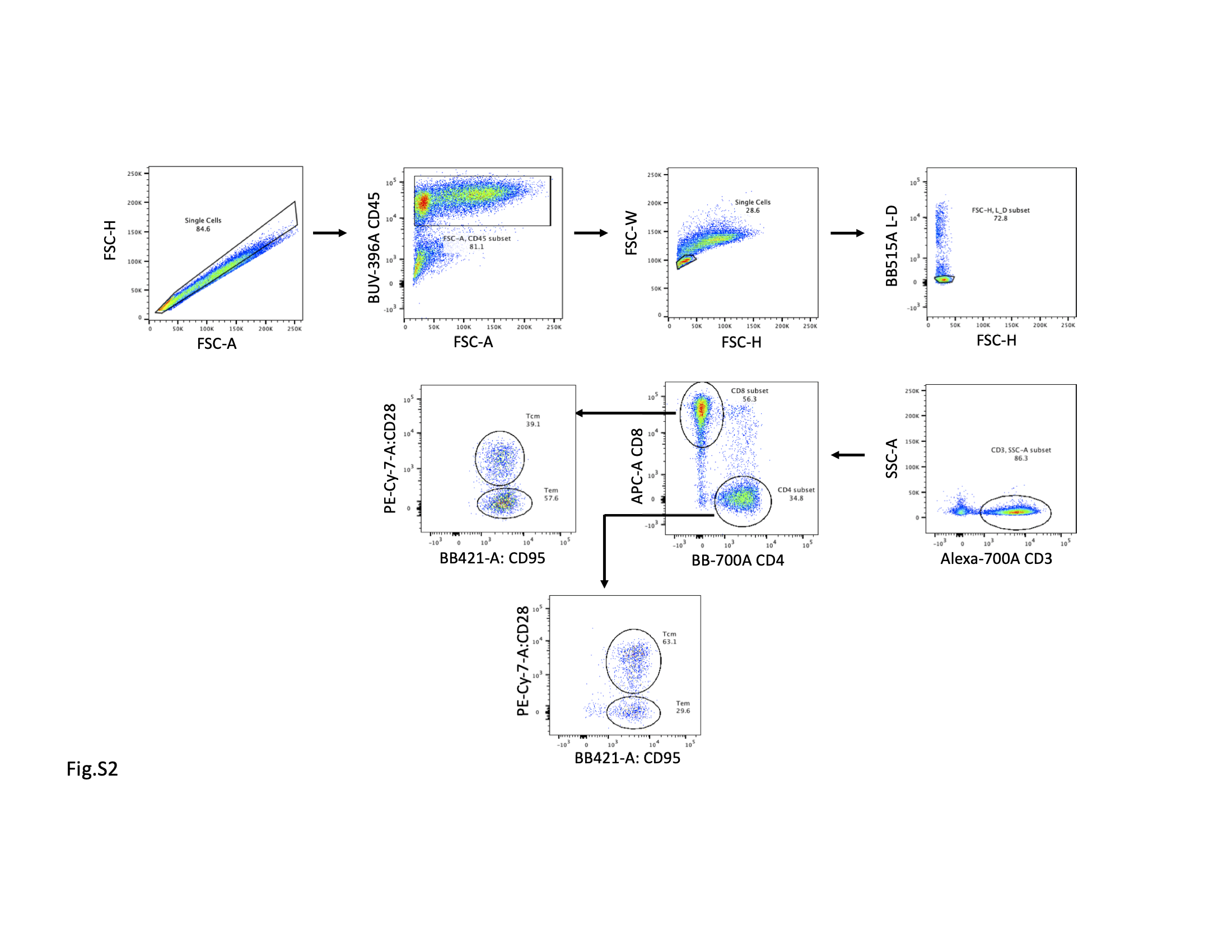

Supplement: Supplementary Figure 2 — Gating strategy for Mtb-specific central memory and effector memory T cell responses. The cells are gated on CD45 and Live/Dead to select live cells and perform red blood cell (RBC) discrimination. This is followed by singlet gating on SSC and FSC -Area, width and Height. Total CD4 and CD8 is then gated on total CD3 population. Central (CD28+CD95+) and effector (CD28-CD95+) memory T cells are then gated on total CD4 and CD8 population in BAL and PBMCs. [file Image_2.tiff]

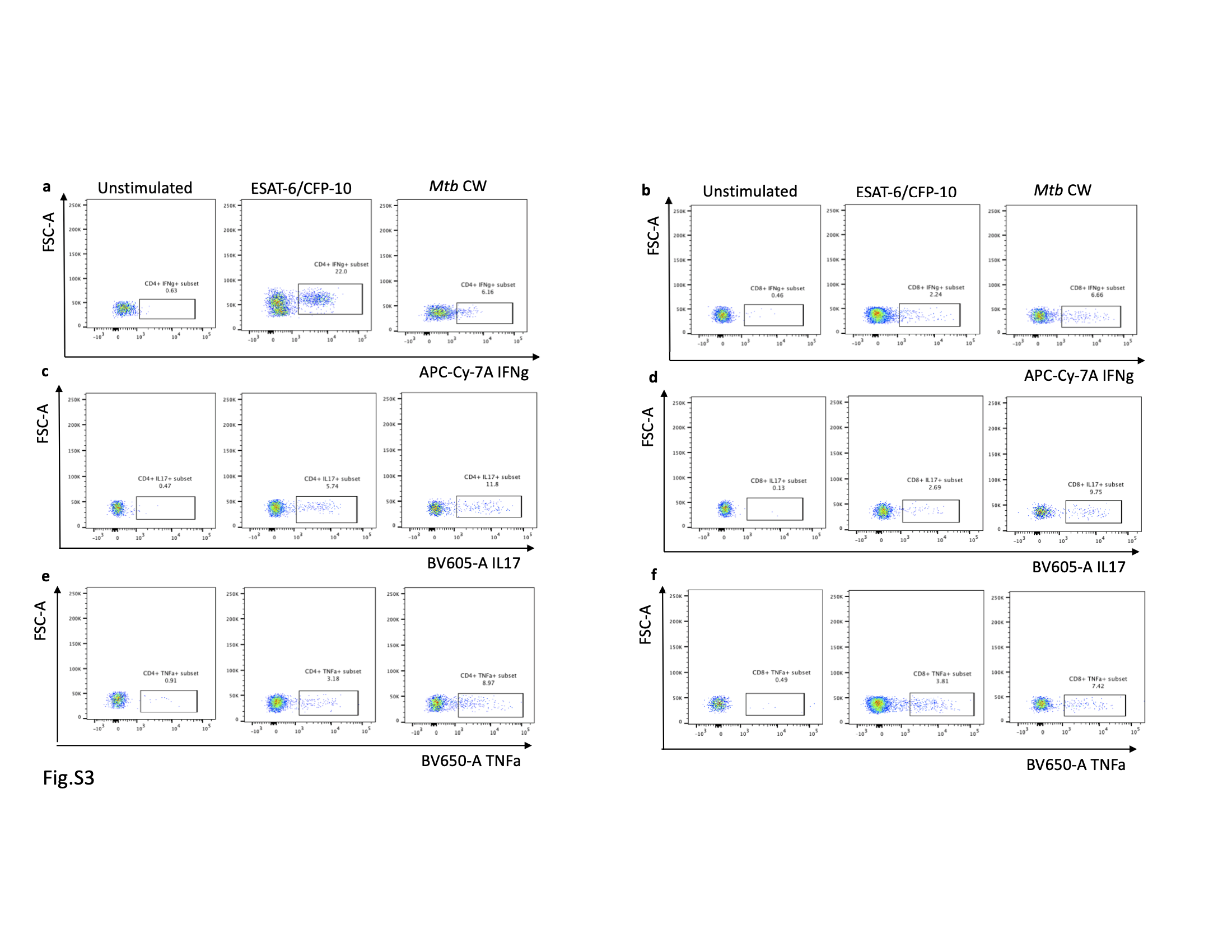

Supplement: Supplementary Figure 3 — Gating strategy for Mtb-specific cytokine positive cells in unstimulated, ESAT-6/CFP-10 stimulated and Mtb CW stimulated BAL samples. (A) CD4+IFN-γ+ T cells (B) CD8+ IFN-γ+ T cells (C) CD4+IL-17+T cells (D) CD8+IL-17+T cells (E) CD4+TNF-α+T cells and (F) CD8+TNF-α+T cells. [file Image_3.tiff]

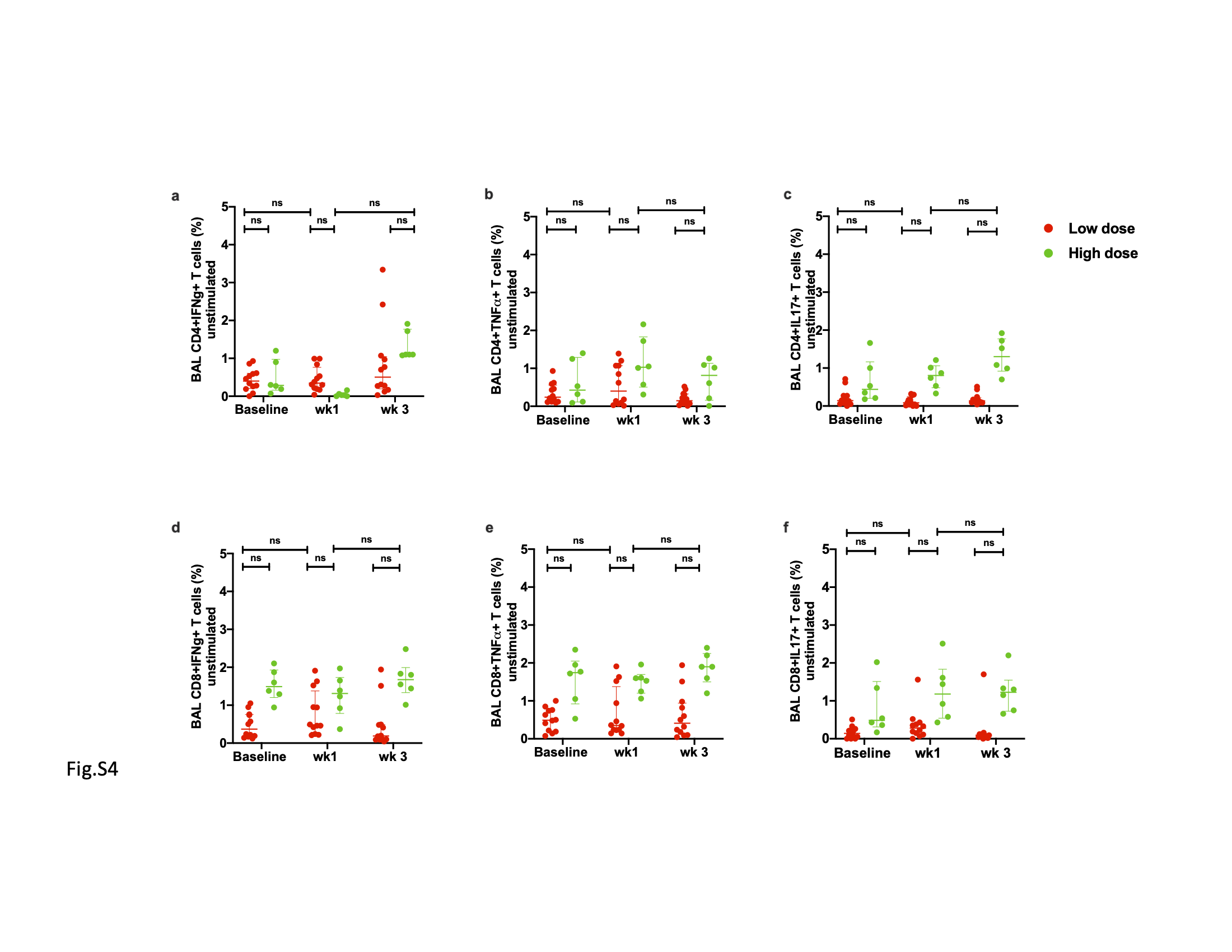

Supplement: Supplementary Figure 4 — Unstimulated responses in BAL of low dose (n = 12) and high dose (n = 6) infected macaques. (A) percentage of CD4+IFN-γ+ T cells, (B) percentage of CD4+TNF-α+ T cells, (C) percentage of CD4+IL-17+ T cells, (D) percentage of CD8+ IFN-γ+ T cells, (E) percentage of CD8+TNF-α+ T cells, (F) percentage of CD8+IL-17+ T cells. The data are expressed as median with interquartile range. *P < 0.05; **P < 0.01; ***P < 0.001; ****P < 0.0001. Significance was determined using Mann Whitney U test in GraphPad Prism v8.4.1. [file Image_4.tiff]

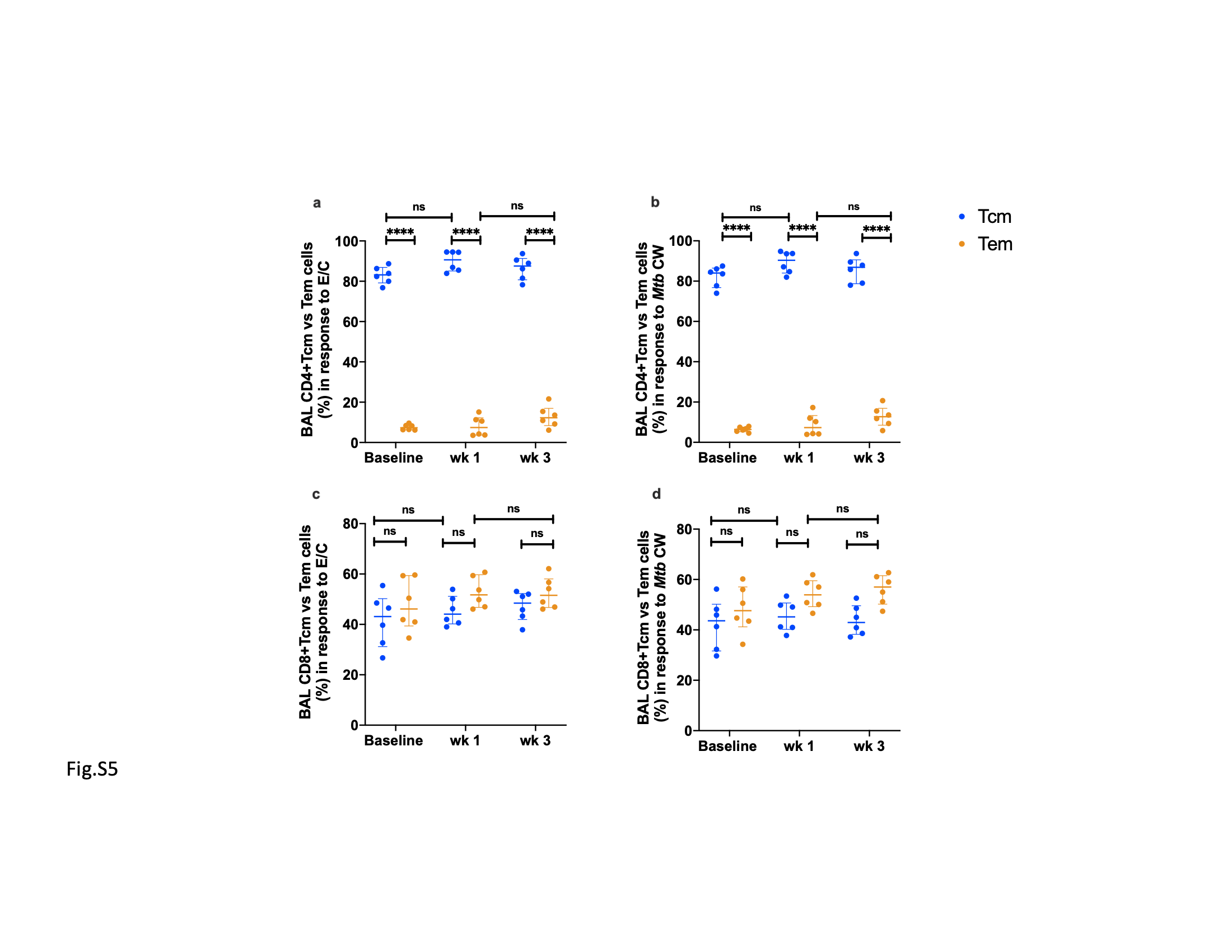

Supplement: Supplementary Figure 5 — Total CD4+ central and Effector memory T cell response in BAL of low dose infection (n = 12). (A) CD4+Tcm and Tem in response to ESAT-6/CFP-10 stimulation, (B) CD4+Tcm and Tem in response to Mtb CW stimulation, (C) CD8+ Tcm and Tem in response to ESAT-6/CFP-10 stimulation. The data are expressed as median with interquartile range. *P < 0.05; **P < 0.01; ***P < 0.001; ****P < 0.0001. Significance was determined using Mann Whitney U test in GraphPad Prism v8.4.1. [file Image_5.tiff]

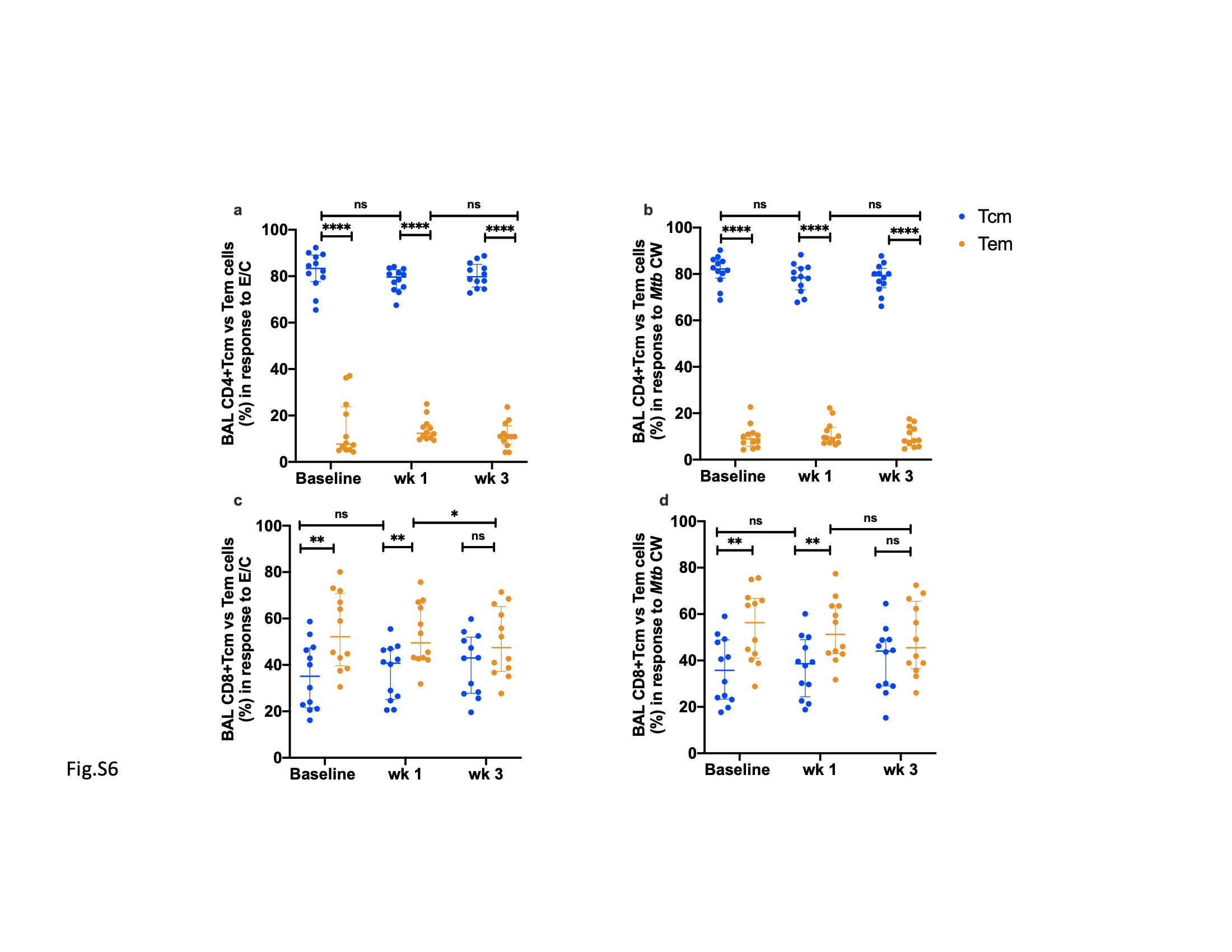

Supplement: Supplementary Figure 6 — Total CD4+ central and Effector memory T cell response in BAL of high dose infection (n = 12). (A) CD4+Tcm and Tem in response to ESAT-6/CFP-10 stimulation, (B) CD4+Tcm and Tem in response to Mtb CW stimulation, (C) CD8+ Tcm and Tem in response to ESAT-6/CFP-10 stimulation and (D) CD8+ Tcm and Tem in response to Mtb CW stimulation. The data are expressed as median with interquartile range. *P < 0.05; **P < 0.01; ***P < 0.001; ****P < 0.0001. Significance was determined using Mann Whitney U test in GraphPad Prism v8.4.1. [file Image_6.tiff]
